# Placebo versus no treatment for people with schizophrenia: A systematic review and meta‐analysis

**DOI:** 10.1002/pcn5.70214

**Published:** 2025-10-08

**Authors:** Nobuyuki Nomura, Yutaro Shimomura, Yuhei Kikuchi, Emily Hird, Hui Wu, Ofer Agid, Stefan Leucht, Hiroyoshi Takeuchi

**Affiliations:** ^1^ Department of Psychiatry and Psychotherapy Technical University of Munich, TUM School of Medicine and Health Munich Germany; ^2^ Department of Neuropsychiatry Keio University School of Medicine Tokyo Japan; ^3^ Deutsches Zentrum für Psychische Gesundheit (DZPG) Partner Site München/Augsburg Munich Germany; ^4^ Department of Psychiatry and Psychotherapy LMU University Hospital, LMU Munich Munich Germany; ^5^ Department of Psychiatry Komagino Hospital Tokyo Japan; ^6^ Institute of Cognitive Neuroscience University College London London UK; ^7^ Schizophrenia Program Centre for Addiction and Mental Health Toronto Ontario Canada; ^8^ Department of Psychiatry University of Toronto Toronto Ontario Canada; ^9^ Institute of Medical Science University of Toronto Toronto Ontario Canada

Antipsychotics are the mainstay of treatment for schizophrenia not only to improve acute symptoms^1^ but also to prevent relapse.^2^ In clinical trials investigating pharmacological interventions, placebo is commonly used as a control to isolate true intervention effects, and it can also improve acute symptoms in people with schizophrenia. The effect of placebo on acute symptoms is smaller than that of antipsychotics, with a small‐to‐moderate effect size (approximately 0.3).^3^, ^4^ By contrast, the “no‐treatment” condition involves participants receiving neither pharmacological treatment (including placebo) nor psychological interventions. Although placebo may be superior to no treatment,^5^ direct comparative evidence in schizophrenia is scarce, and it remains unclear whether placebo provides symptom improvement or relapse prevention over no treatment.

To address this critical gap, we conducted a systematic review and meta‐analysis with two objectives: (1) to assess the volume and temporal distribution of evidence comparing placebo with no treatment in people with schizophrenia, and (2) if sufficient data were available, to examine whether a placebo offers any therapeutic advantage over no treatment. We systematically searched multiple databases, including CENTRAL, MEDLINE, Embase, PsycINFO, and clinical trial registries, up to March 20, 2025. The protocol was registered in the Cochrane Database of Systematic Reviews.^6^ We identified randomized controlled trials (RCTs) explicitly comparing placebo with no treatment among individuals with schizophrenia spectrum disorders.

Our initial search was conducted on January 13, 2023, and updated on March 20, 2025, yielding 7659 records. After duplicate removal, we screened 6250 records, and ultimately included only three RCTs, highlighting the lack of evidence.^7^, ^8^, ^9^ Notably, all were conducted between 1956 and 1957, underscoring the absence of contemporary research on this question. Each compared an antipsychotic with both placebo and no treatment. Pearl et al.^7^ randomized 150 male inpatients to reserpine, placebo, or no drug for a 2‐month evaluation. Penman allocated 80 male chronic inpatients across five arms within a 6‐month program that included both control conditions.^8^ Sibilio et al. enrolled 93 female long‐stay inpatients in a 4‐month rotating design,^9^ from which we analyzed only the initial 28‐day comparison of placebo and no treatment. Of the three identified studies, only two provided analyzable data, enabling assessment of only two critical outcomes.^8^, ^9^ The risk of study discontinuation in the placebo group (*n* = 2, *N* = 47) compared to the no‐treatment group (*n* = 4, *N* = 47) was estimated as risk ratio (RR) = 0.50 (95% confidence interval [CI] [0.11, 2.35], *p* = 0.38; heterogeneity = not applicable) and the risk for mortality between placebo (*n* = 0, *N* = 47) and no‐treatment (*n* = 1, *N* = 47) was estimated as RR = 0.33 (95% CI [0.01, 7.62], *p* = 0.49; heterogeneity = not applicable) (Figure 1).

**Figure 1:**
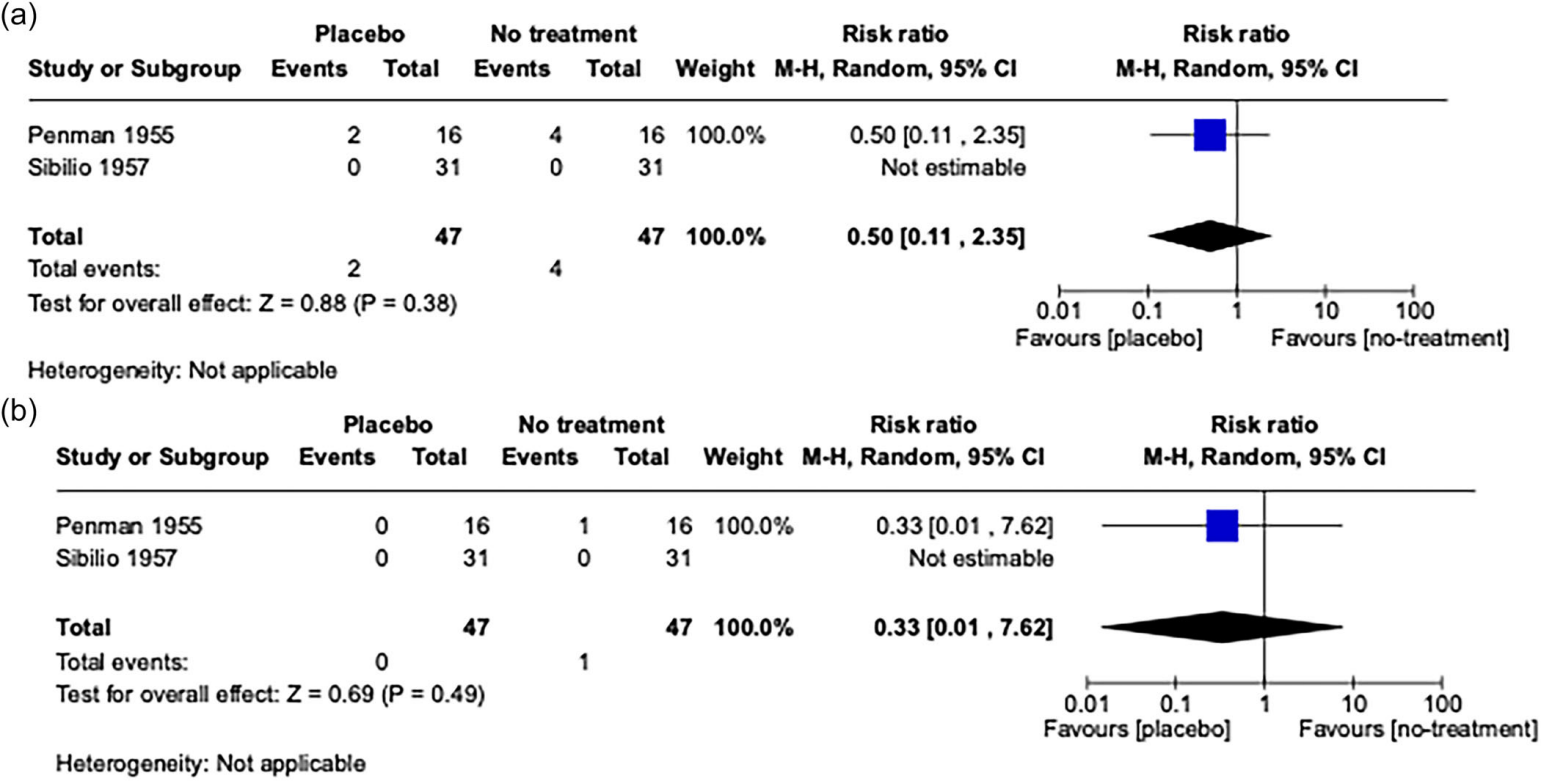
Forest plot for (a) risk of study discontinuation and (b) risk of mortality. CI, confidence interval.

Our review identified only three trials from the 1950s that compared placebo with no treatment in people with schizophrenia. For our first objective, which was to assess the volume and temporal distribution of evidence, we found that the evidence base is sparse and outdated, with no later decades contributing additional trials. The complete absence of modern trials likely reflects ethical concerns about withholding treatment. Nonetheless, without a no‐treatment comparator, placebo effects cannot be separated from spontaneous change or observation effects, limiting interpretation of placebo‐controlled trials. For our second objective, which was to examine whether placebo offers any therapeutic benefit over no treatment, the available data were too limited to yield a precise estimate. Across two short trials, we observed no substantial difference between placebo and no‐treatment groups in premature trial withdrawal or in all‐cause mortality. However, a previous meta‐regression has shown that placebo response has increased over time, suggesting that these early trials may underestimate the current magnitude of placebo effects.^10^ Therefore, more recent data including untreated groups are needed to clarify the effects of placebo compared to no treatment. Given the ethical challenges of randomizing patients to no‐treatment arms, future studies should adopt ethically acceptable designs, such as naturalistic cohorts and mirror image within‐subject analyses, to compare outcomes during a placebo period with those during a no‐treatment period. Moreover, previous meta‐analyses have shown that placebo effects are influenced by patient expectations, clinician–patient interactions, and structured trial settings.^3^, ^4^ Further research should also examine factors influencing placebo responsiveness. In conclusion, there are currently no adequate data to examine placebo effects in this context, nor is there robust evidence comparing placebo with no treatment in schizophrenia. Future studies using ethically acceptable designs are needed to determine whether placebo confers any therapeutic benefit over no treatment in people with schizophrenia.

## AUTHOR CONTRIBUTIONS

Nobuyuki Nomura, Yutaro Shimomura, Yuhei Kikuchi, Emily Hird, Hui Wu, Ofer Agid, Stefan Leucht, and Hiroyoshi Takeuchi contributed to the development of the protocol. Nobuyuki Nomura, Yutaro Shimomura, and Yuhei Kikuchi screened studies, extracted data, and conducted the analyses. Stefan Leucht and Hiroyoshi Takeuchi supervised the overall conduct of the review. The manuscript was primarily drafted by Nobuyuki Nomura and Hiroyoshi Takeuchi. All authors reviewed and approved the final version of the manuscript.

## CONFLICT OF INTEREST STATEMENT

The authors declare no conflicts of interest.

## ETHICS APPROVAL STATEMENT

N/A.

## PATIENT CONSENT STATEMENT

N/A.

## CLINICAL TRIAL REGISTRATION

N/A.

## Data Availability

The data that support the findings of this study are available from the corresponding author upon reasonable request. All data used in this review were extracted from published articles.
